# Fossil Collection at the Zoology Museum of the University of Concepción: enhancing understanding of Chile's past biodiversity

**DOI:** 10.3897/BDJ.12.e117275

**Published:** 2024-02-29

**Authors:** Francisca Alejandra Beltrán Echeverría, Laura Tavera Martínez, Cristián E. Hernández

**Affiliations:** 1 Museo de Zoología, Departamento de Zoología, Facultad de Ciencias Naturales y Oceanográficas, Universidad de Concepción, Casilla 160- C, Concepción, Chile Museo de Zoología, Departamento de Zoología, Facultad de Ciencias Naturales y Oceanográficas, Universidad de Concepción, Casilla 160- C Concepción Chile; 2 Postdoctoral Research, Departamento de Zoología, Facultad de Ciencias Naturales y Oceanográficas, Universidad de Concepción, Concepción, Chile Postdoctoral Research, Departamento de Zoología, Facultad de Ciencias Naturales y Oceanográficas, Universidad de Concepción Concepción Chile; 3 Departamento de Zoología, Facultad de Ciencias Naturales y Oceanográficas, Universidad de Concepción, Concepción, Chile Departamento de Zoología, Facultad de Ciencias Naturales y Oceanográficas, Universidad de Concepción Concepción Chile; 4 Laboratorio de Ecología Evolutiva y Filoinformática, Departamento de Zoología, Facultad de Ciencias Naturales y Oceanográficas, Concepción, Chile Laboratorio de Ecología Evolutiva y Filoinformática, Departamento de Zoología, Facultad de Ciencias Naturales y Oceanográficas Concepción Chile

**Keywords:** fossils, databases, biological collections, Chile, fossiliferous localities, marine invertebrates.

## Abstract

**Background:**

The digital inventory of paleontological material stored in Chilean museums is highly relevant as it increases accessibility to information, both locally and over long distances, while reducing wear and tear on specimens caused by physical manipulation. The Fossil Collection database of the Museum of Zoology of the University of Concepción (UCC_MZUC_FOS) includes 144 records, with the main representatives being marine invertebrates of the Bivalvia, Echinoidea and Gastropoda classes. Notable species include *Encopecalderensis*, *Hemiasterwayensis*, *Zygochlamyspatagonica* and *Retrotapesexalbidus*, most of which come from important Chilean fossil sites. Material was collected between 1970 and 2017, with a large portion of it being donated and identified by Professor Emeritus Hugo I. Moyano and Dr. Alberto Larraín. Although the specimens contained in the resource offer basic collecting information, they substantially contribute to sharing knowledge on the fossils kept in the museums throughout the country, while providing data on their distribution.

**New information:**

This resource corresponds to the first publication of data on faunal fossils from a museum collection in Chile on the Global Biodiversity Information Facility (GBIF) platform, thereby enhancing the understanding and documentation of Chile's paleontological heritage and its national biodiversity.

## Introduction

Chile’s palaeontological heritage is rich and diverse, with numerous fossiliferous localities distributed throughout its territory (Vega-Jorquera et al. 2015). These sites cover different geological periods and represent a wide range of paleoenvironments. This natural and scientific heritage, which is crucial for researching and understanding past biodiversity, must be protected and preserved. Accredited institutions, such as universities and museums, play a vital role in safeguarding this heritage by creating biological and scientific collections. This ensures its long-term preservation and accesibility for research inquiries.

The creation of inventories of biological collections and their subsequent digitisation in standard biodiversity formats, such as Darwin Core (DwC), has become increasingly relevant in recent years. This format provides a common language for sharing biodiversity data through a set of terms with clearly defined semantics that can be understood by people or interpreted by software, allowing the appropriation of the encoded data (Wieczorek et al. 2012). Access to local, regional and global information on specimens is available directly from a computer, cell phone or device with an internet connection, free of charge, eliminating or reducing the need of travelling to consult collections or handle specimens, thereby preventing wear and tear caused by physical handling. In addition, the information associated with the specimen, such as habitat, environmental variables and biological associations, provided by the databases, favours research in different areas of knowledge, such as evolution, systematics and ecology (Lendemer et al. 2020).

Given the importance of faunal fossil records in Chile, the objective of this work is to contribute to the knowledge and documentation of fossils housed in Chilean museums, with emphasis on the material from localities of the national territory. The Fossil Collection at the Zoology Museum of the University of Concepción (UCCC_MZUC_FOS) is mainly composed of material bequeathed by the professors of the Faculty of Natural Sciences and Oceanography, Hugo I. Moyano and Alberto Larraín, starting in 1986. The collection consists of 144 specimens of fossil fauna that provide information on two important geological formations of the Biobío Region, the Quiriquina Formation and the Tubul Formation, as well as fossiliferous localities in the regions of Antofagasta (Quebrada El Way) and Atacama (Punta Cabeza de Vaca), amongst others.

This collection is digitised in DwC format and published in the Global Biodiversity Information System–GBIF, constituting the first database on faunal fossils in Chile. Therefore, this work is crucial for the preservation and accesibility of Chile's paleontological heritage.

### Geological setting

The Quebrada El Way fossiliferous deposit is located about 10 km south of the City of Antofagasta and is the type locality of the El Way Formation (Wenzel 1957), a succession of marine strata composed mainly of calcilutites, calcarenites and limestones. Its deposits belong to the Lower Cretaceous (Brüggen 1950, Wenzel 1957, Harrington 1961, Barceló 1972). The specimens housed at the Zoology Museum of the University of Concepción (MZUC-UCCC) that come from this formation correspond to *Hemiasterwayensis*, a species described by Larraín (1985).

The Quiriquina Formation represents Maastrichtian (Upper Cretaceous) transgressive deposits (Biró-Bagóczky 1982) that outcrop from Algarrobo, San Antonio Province, to Lebu, Arauco Province. The type locality of the formation is located in Bahía Las Tablas, Quiriquina Island and the parastratotype locality is in Cocholgüe, north of Tomé. The strata of the Quiriquina Formation were deposited in a gradually deepening coastal environment (Palma-Heldt and Quinzio 2006). This formation is known for their vast faunal record, including ammonoids, nautiloids, bivalves, gastropods, scaphopods, elasmobranch teeth and skeletal remains of marine reptiles, which have been studied by several scientists since the 1840s, including (Darwin 1846, Gay 1848, Gay 1854, Philippi 1887, Casamiquela 1969, amongst others). The latest specimen described from this formation is the plesiosaur *Aristonectesquiriquinensis* (Otero et al. 2015). The specimens in the MZUC-UCCC Fossil Collection that come from this formation are dental structures of *Carcharias* sp. and *Plesiosaurus* sp., *Baculites* sp., *Dentalium* sp. and shells of *Pacitrigonia* sp., amongst others.

The specimens of the species *Encopecalderensis* listed in the collection database come from the upper levels of a coastal cliff south of Punta Cabeza de Vaca, Atacama Region, which is comprised of Pliocene marine sediments (Covacevich and Frassinetti 1977).

Finally, the Tubul Formation, whose type locality is located in the southern sector of the Gulf of Arauco, was deposited rapidly during the late Pliocene near the Plio-Pleistocene boundary in a nearshore area (Pineda 1983, Pineda 1986). This marine assemblage contains a wide diversity of invertebrates, including the bivalves *Zygochlamyspatagonica* and *Retrotapesexalbidus*.

## Geographic coverage

### Description

**Figure 1.**
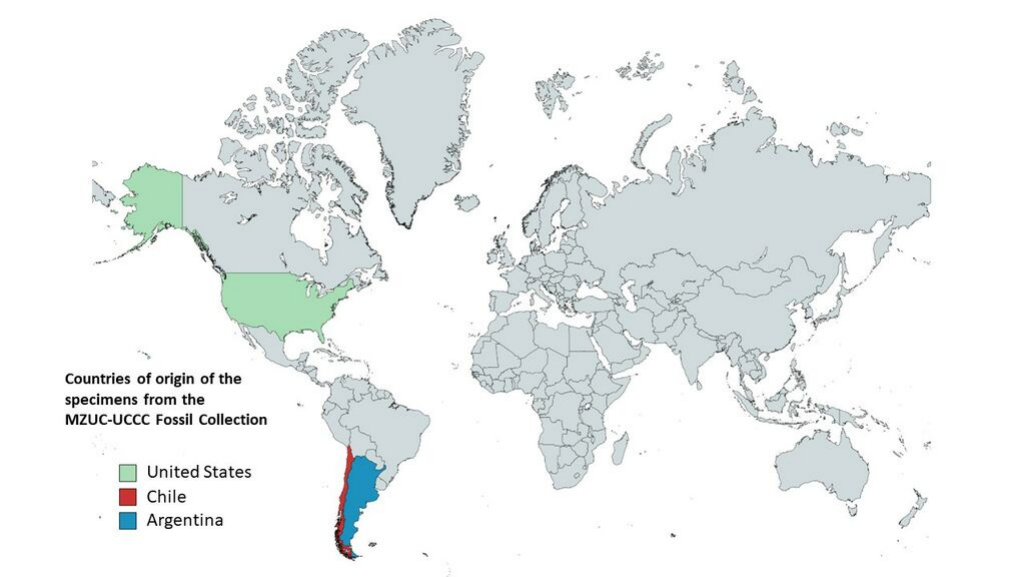
World distribution map of the specimens from the Fossil Collection of the Museum of Zoology of the University of Concepción (MZUC-UCCC).

All the specimens come from the American continent, 141 are from Chile, two from Argentina and a single specimen from the United States (Fig. 1). Two sampling localities were found outside the national territory: Bristol Bay, in the United States and Cerro Negro, in Argentina. The fossil material recovered in Chile comes from localities distributed from north to south of the country, including the regions of Antofagasta, Atacama, Biobío, Los Ríos, Los Lagos, Magallanes and Chilean Antarctica (Table 1). None of the specimens presents geographical coordinates and many of them come from unspecific localities that cannot be represented on a map, but when the geological formation is detailed, it is posible to deduce their region of origin. In addition, the collection data on the museum labels do not present information on the sampling methods used in the extraction of the specimens that compose the present database.

## Taxonomic coverage

### Description

**Figure 2.**
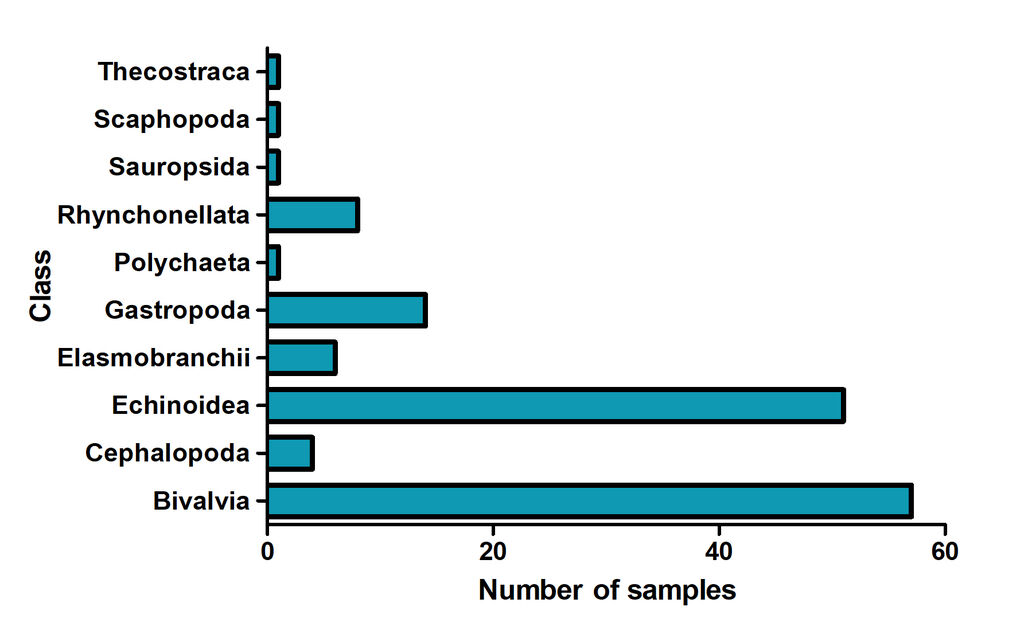
Number of specimens for each class registered in the MZUC-CCC Fossil collection.

**Figure 3.**
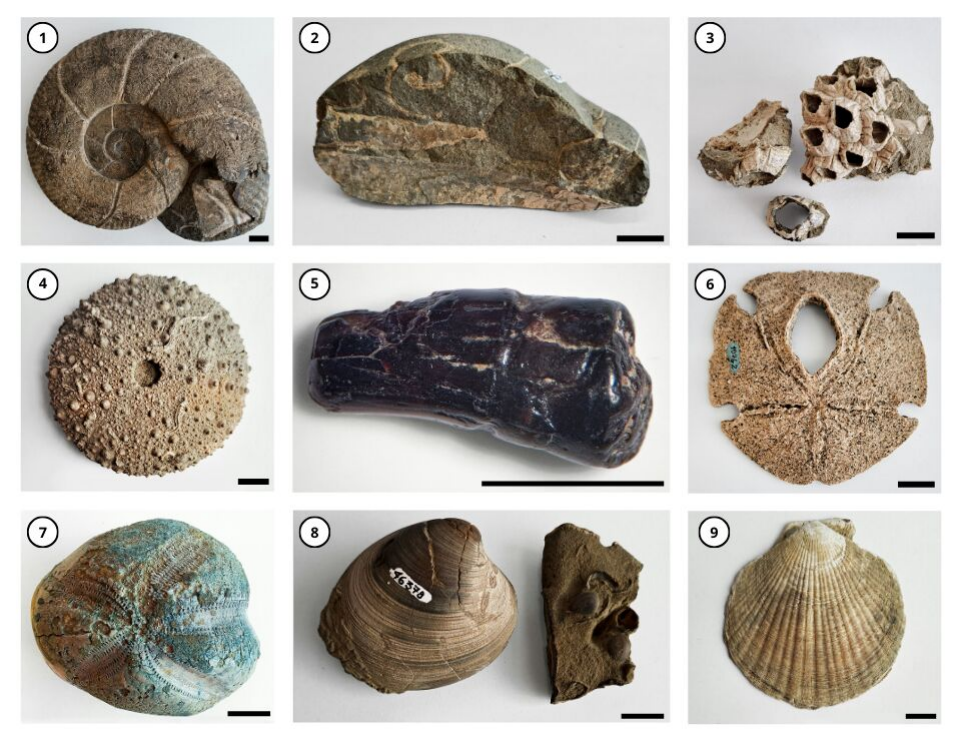
Representatives of the MZUC-UCCC Fossil collection. **1**: Cephalopoda MZUC-UCCC 45745. **2**: *Baculites* sp. MZUC-UCCC 46366. **3**: *Balanus* sp. MZUC-UCCC 45746. **4**: *Loxechinus* sp. MZUC-UCCC 18133. **5**: *Plesiosaurus* sp. MZUC-UCCC 45747. **6**: *Encopecalderensis* MZUC-UCCC 10762. **7**: *Hemiasterwayensis* MZUC-UCCC 10811. **8**: *Retrotapesexalbidus* MZUC-UCCC 45722. **9**: *Zygochlamyspatagonica* MZUC-UCCC 45736. Scale: 1 centimetre. Photographs by: Francisca Beltrán.

All taxa were identified to the lowest possible taxonomic category. The taxonomic coverage included one kingdom, six phyla, 10 classes, 24 orders, 34 families, 42 genera and 25 species (valid and invalid). The most represented classes in the collection are Bivalvia, with 57 specimens and Echinoidea, with 51 specimens. They are followed by the classes Gastropoda (14), Rhynchonellata (8), Cephalopoda (4), Polychaeta (1), Sauropsida (1), Scaphopoda (1) and Thecostraca (1) (Fig. 2). The main representatives of this collection correspond to marine invertebrates, such as *Encopecalderensis*, *Hemiasterwayensis*, *Zygochlamyspatagonica* and *Retrotapesexalbidus*, amongst others (Fig. 3).

**Data quality control**: The taxonomic information originally recorded by the collectors and/or determiners of each specimen was validated. For this, the “Match Taxa” tool of the World Register of Marine Species (WoRMS Editorial Board 2022) was used, followed by the “Species matching” tool of the Global Biodiversity Information Facility (GBIF.org 2022). These tools made it possible to update currently-accepted species and correct scientific names with misspellings. These changes were incorporated both in the DwC databases and in the labels of each specimen. Finally, validation of the collection database in DwC format was carried out using the GBIF data validator. This filtering of the information was carried out prior to the publication of the resource in the GBIF platform, verifying that the structure of the data complied with the criteria of the DwC format. This ensured that the data were published with good quality, guaranteeing that the published information was accurate and reliable.

### Taxa included

**Table:** 

Rank	Scientific Name	Common Name
class	Bivalvia	Bivalve
class	Cephalopoda	Cephalopod
genus	*Baculites*	
genus	*Balanus*	Acorn barnacle
genus	*Carcharias*	
genus	*Dentalium*	
genus	*Fusitriton*	
genus	*Loxechinus*	
genus	*Ostrea*	Oyster
genus	*Pacitrigonia*	
genus	*Plesiosaurus*	Plesiosaur
genus	*Retrotapes*	Clam
genus	*Rhynchonella*	
genus	*Scapanorhynchus*	
genus	*Schizaster*	
genus	*Serpula*	
genus	*Tegula*	
genus	*Trophon*	
genus	*Turritella*	
species	*Acanthinaunicornis*	
species	*Belapaessleri*	
species	*Chorusgiganteus*	Trumulco snail
species	*Cirsotremamagellanicum*	
species	*Crepiduladilatata*	
species	*Echinarachniusparma*	Sand dollar
species	*Encopecalderensis*	
species	*Ennuculagrayi*	
species	*Ensismacha*	Concha navaja, huepo, Chilean macha, navajuela
species	*Eurhomaleaexalbida*	Clam
species	*Euspiraguamblinensis*	
species	*Fusitritonmagellanicus*	
species	*Hemiasterwayensis*	
subspecies	*Iheringiellapatagonensis*	
species	*Ischyrhizachilensis*	Plesiosaur
species	*Leukomaantiqua*	Clam
species	*Macoplomainornata*	
species	*Magellaniavenosa*	
species	*Mangeliapaessleri*	
species	*Monophorasterdarwini*	
species	*Pandorabraziliensis*	
species	*Pseudechinusmagellanicus*	Dwarf hedgehog
species	*Tindariopsissulculata*	
species	*Xymenopsisdispar*	
species	*Zygochlamyspatagonica*	Patagonian oction

## Temporal coverage

**Data range:** 1970-4-30 – 2017-8-23.

### Notes

**Figure 4.**
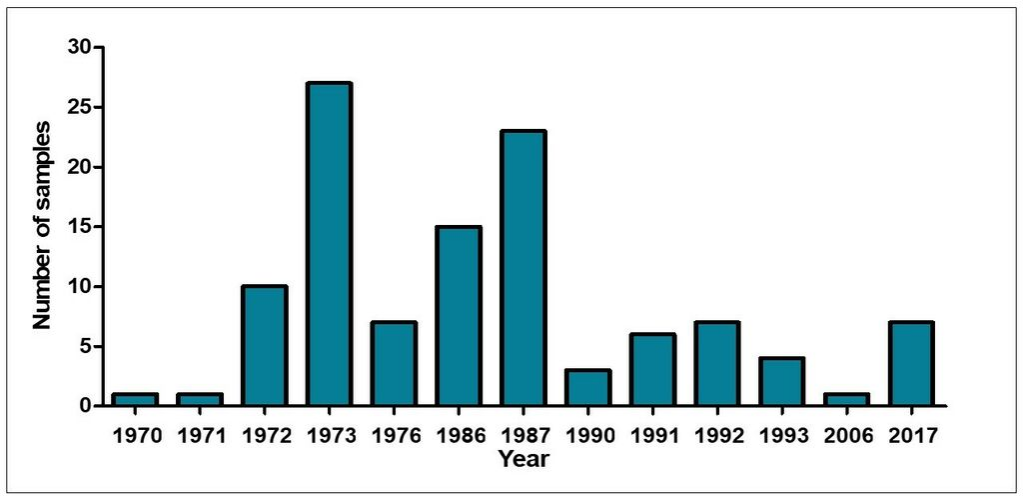
Number of specimens from the MZUC-UCCC Fossil Collection collected for each recorded sampling year.

The specimens were collected between 1970 and 2017 (Fig. 4) and were deposited with other collections of the Museum of Zoology of the University of Concepción, mainly the Echinoderm Collection and the Mollusk Collection. Subsequently, between 2017 and 2022, the MZUC-UCCC Fossil Collection was formed.

## Usage licence

### Usage licence

Creative Commons Public Domain Waiver (CC-Zero)

### IP rights notes

This work is licensed under a Creative Commons Attribution Non-Commercial (CC-BY-NC) 4.0 License.

## Data resources

### Data package title

Fossil Collection of the Zoology Museum of the University of Concepción UCCC_MZUC_FOS

### Resource link

https://doi.org/10.15468/wdmeh2

### Number of data sets

1

### Data set 1.

#### Data set name

Fossil Collection of the Zoology Museum of the University of Concepción UCCC_MZUC_FOS

#### Data format

Darwin Core

#### Download URL

https://www.gbif.org/dataset/c83ef5fa-e70f-460d-80cc-435694892a6a

#### Description

The resource consists of a database of fossils belonging to the Zoology Museum of the University of Concepción (Beltrán-Echeverría et al. 2023). Most of the specimens were collected by Emeritus Professor Hugo Moyano and Dr. Alberto Larraín. The database includes a total of 144 records associated with a number MZUC-UCCC, mainly from Chile and, to a lesser extent, from Argentina and the United States. The main Chilean localities include the Quiriquina Formation and Tubul Formation, from the Upper Cretaceous and the Plio-Pleistocene of the Biobío Region, respectively, Quebrada El Way from the Lower Cretaceous of the Antofagasta Region and Punta Cabeza de Vaca, from the Pliocene of the Atacama Region.

The following data categories from the Darwin Core Standard were used:

**Data set 1.** 

Column label	Column description
occurrenceID	Unique correlative indicator of the biological record.
basisOfRecord	“FossilSpecimen” for all records.
type	“PhysicalObject” for all records.
institutionCode	“Museum of Zoology of the University of Concepción (MZUC-UCCC)” for all records.
institutionID	The identifier of the institution to which the resource was referred.
collectionCode	“UCCC_MZUC_FOS” for all records.
collectionID	The identifier of the collection or dataset to which the resource was derived.
catalogNumber	Correlative number.
datasetName	“Fossil Collection of the Zoology Museum of the University of Concepción” for all records.
language	Spanish.
license	CC BY-NC 4.0.
rightsHolder	“Zoology Museum of the University of Concepción” for all records.
accessRights	“not-for-profit use only” for all records.
ownerInstitutionCode	“Museum of Zoology of the University of Concepción (MZUC-UCCC)” for all records.
recordedBy	Name of the person responsible for the registration.
individualCount	Number of registered individuals.
organismScope	DwC instance type description: Organism. It can be used to indicate whether the dwc instance: Organism represents a discrete organism or whether it represents a particular type of aggregation.
previousIdentifications	A list of previous naming assignments to the dwc: Organism.
preparation	“Fossil” for all records.
disposition	“In collection” for all records.
eventDate	The date and time or interval during which the event occurred.
year	The four-digit year in which the event occurred, according to the Common Era Calendar.
month	The entire month in which the event occurred.
day	The entire day of the month in which the event occurred.
verbatimEventDate	The original textual representation of the date and time information for the event.
fieldNumber	An identifier given to the event in the field.
continent	The name of the continent on which the locality occurs.
waterBody	The name of the body of water in which the locality occurs.
islandGroup	The name of the group of islands on which the locality occurs.
island	The name of the island on or near which the locality occurs.
country	The name of the country or main administrative unit in which the locality occurs.
countryCode	The standard code for the country in which the locality occurs.
stateProvince	The name of the next administrative region smaller than the country (state, region) in which the locality occurs.
county	The full, unabbreviated name of the administrative region next smaller than stateProvince in which the locality occurs.
municipality	The full, unabbreviated name of the administrative region smaller than the county in which the locality occurs.
locality	The specific description of the place.
verbatimLocality	The original textual description of the place.
verbatimDepth	The original description of the depth below the local surface.
locationRemarks	Comments or notes from the locality.
earliestEonOrLowestEonothem	The full name of the earliest possible geochronological eon attributable to the stratigraphic horizon from which the element was collected.
latestEonOrHighestEonothem	The full name of the latest possible geochronological eon attributable to the stratigraphic horizon from which the element was collected.
earliestEraOrLowestErathem	The full name of the earliest possible geochronological era attributable to the stratigraphic horizon from which the element was collected.
latestEraOrHighestErathem	The full name of the latest possible geochronological era attributable to the stratigraphic horizon from which the feature was collected.
earliestPeriodOrLowestSystem	The full name of the earliest possible geochronological period attributable to the stratigraphic horizon from which the catalogued element was collected.
latestPeriodOrHighestSystem	The full name of the latest possible geochronological period attributable to the stratigraphic horizon from which the feature was collected.
earliestEpochOrLowestSeries	The full name of the earliest possible geochronological epoch attributable to the stratigraphic horizon from which the element was collected.
latestEpochOrHightestSeries	The full name of the latest possible geochronological epoch attributable to the stratigraphic horizon from which the feature was collected.
earliestAgeOrLowestStage	The full name of the earliest possible geochronological age attributable to the stratigraphic horizon from which the element was collected.
latestAgeOrHighestStage	The full name of the latest possible geochronological age attributable to the stratigraphic horizon from which the element was collected.
lithostratigraphicTerms	The combination of all lithostratigraphic names of the rock from which the element was collected.
formation	The full name of the lithostratigraphic formation from which the element was collected.
identifiedBy	A list of names of people, groups or organisations that assigned the name to the taxon.
dateIdentified	The date the taxon was determined.
identificationReferences	A list of references (publication) used in identification.
typeStatus	Nomenclatural types (holotype, paratype).
scientificName	The name of the species or taxon of the record.
scientificNameAuthorship	Authorship information for the scientific name.
kingdom	The scientific name of the kingdom in which the taxon is classified.
phylum	The scientific name of the phylum in which the taxon is classified.
class	The scientific name of the class in which the taxon is classified.
order	The scientific name of the order in which the taxon is classified.
family	The scientific name of the family in which the taxon is classified.
subfamily	The scientific name of the subfamily in which the taxon is classified.
genus	The scientific name of the genus in which the taxon is classified.
specificEpithet	The name of the species epithet of the scientific name.
taxonRank	The taxonomic rank of the most specific name of the scientific name.
verbatimTaxonRank	The taxonomic rank of the most specific name of the scientific name as it appears in the original record.
vernacularName	Common name.
taxonomicStatus	The status of use of the scientific name (invalid, synonym, valid).
acceptedNameUsage	Accepted name in use.

## Additional information

### Contributions made to the MZUC-UCCC Fossil collection

Prior to digitising the collection, several researchers and students reviewed the collection, mainly from the Faculty of Natural and Oceanographic Sciences of the University of Concepción. The marine biologist Marina Fuentes created the Fossil Collection of the Zoology Museum of the University of Concepción in 2017, performing the verification of the status of the specimens, their sampling data and taxonomic determination. Dr. Alberto Larraín Prat collected and determined several of the specimens and authored the species *H.wayensis*, whose holotype is in this collection. Professor Emeritus Hugo Moyano also collected and determined some specimens. Finally, in 2022, the resource was created by the author, optimising the internal database of the Museum of Zoology according to the standard criteria of the Darwin Core format reported in this study, along with the development of the metadata and the photographic record of the specimens of the collection.

### Conclusions

This resource is the first publication on faunal fossil data from a museum collection in Chile, thus constituting a valuable contribution to the knowledge of historical biodiversity. It is located in one of the most important international repositories of biodiversity (GBIF), with free access for the community and with a standard format (DwC) that facilitates its understanding. For this reason, the digitisation and publication of biological (specifically, paleontological) collections in formats accepted worldwide, are of great relevance to expand access to information and promote the development of research in different areas of biology, thus allowing us to understand the changes of past biodiversity through large temporal and spatial scales.

## Figures and Tables

**Table 1.** Main collection locations for specimens from the MZUC-UCCC Fossil Collection.

**Location**	**Country**	**Region (if applicable)**
Bristol Bay	United States	
Cerro Negro, Potrerillos	Argentina	
Checo del Cobre, Nantoco	Chile	Atacama
Punta Cabeza de Vaca	Chile	Atacama
Quebrada El Way	Chile	Antofagasta
Cocholgüe	Chile	Biobio
Lirquén	Chile	Biobio
Las Tablas Beach, Quiriquina Island	Chile	Biobio
Quiriquina Formation	Chile	Biobio
Caleta Tubul	Chile	Biobio
Tubul Formation	Chile	Biobio
Arauco Beach	Chile	Biobio
Arauco to Lebu road	Chile	Biobio
Road to Lebu	Chile	Biobio
Valdivia	Chile	Los Ríos
Cuesta Los Quinientos	Chile	Los Ríos
Quenuir, Maullín River mouth	Chile	Los Lagos
Sector Flamenco, between Caleta San Sebastián and Bahía Inútil	Chile	Magallanes and Chilean Antarctica
